# Influenza epidemic model using dynamic social networks of individuals with cognition maps

**DOI:** 10.1016/j.mex.2020.101030

**Published:** 2020-08-19

**Authors:** Leonardo López, Maximiliano Fernández, Leonardo Giovanini

**Affiliations:** a Barcelona Institute for Global Health, Barcelona, Spain; b Research Institute for Signals, Systems and Computational Intelligence, sinc(i), FICH-UNL-CONICET, Argentina

**Keywords:** Individual-based model, Cellular automata, Fuzzy-cognitive maps, Infectious disease

## Abstract

The dynamic of infectious disease is the result of the interplay between the spread of pathogens and individuals’ behaviour. This interaction can be modelled through a network of interdependent dynamical blocks with multiple feedback connections. Epidemic outbreaks trigger behavioural responses, at the group and individual levels, which in turn influence the development of the epidemic. The interactions can be modelled through adaptive temporal networks whose nodes represent the individuals interconnected. Here we introduce an individual-based model where the behaviour of each agent is governed by its appreciation of the environment and external stimulus and its appreciation of its environment. It is built as a combination of three interacting blocks: (i) individual behaviour, (ii) social behaviour and (iii) health state.

• Here, we introduce an individual-based model.
• Individual's behaviour is modelled through the interplay of information of its health state as well as its neighbourhood (infected and recovered neighbours) and global epidemic situation;
• Social behaviour is modelled through contact network that aggregates the behaviour and health state of the individuals;
• The proposed model allows to use a wide range of alternatives for modelling each of these blocks, that provides flexibility to select the most adequate tool to model each component of the framework.

## Graphical abstract

**Figure:**
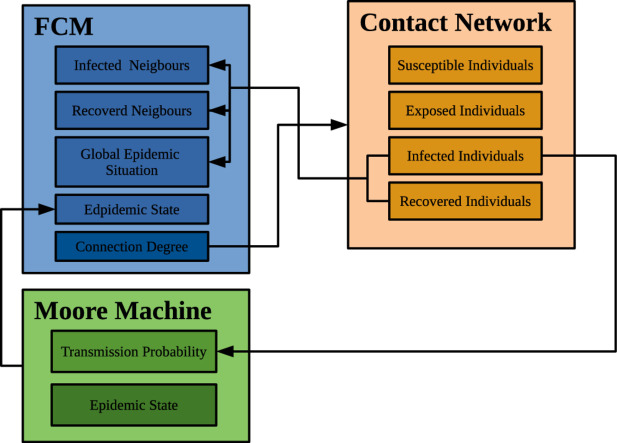

Specifications table

**Table:** 

Subject Area	Computer Science
More specific subject area	*Computational Modelling*
Name and reference of original method	Addressing population heterogeneity and distribution in epidemics models using a cellular automata approach. DOI: 10.1186/1756-0500-7-234 Individual Decision Making Can Drive Epidemics: A Fuzzy Cognitive Map Study. DOI: 10.1109/TFUZZ.2013.2251638
Resource availability	https://github.com/LeonardoL87/IBM-Model-for-Influenza.-Adaptive-network-using-Celular-Automata.git

## Method details

**Fig. 1:**
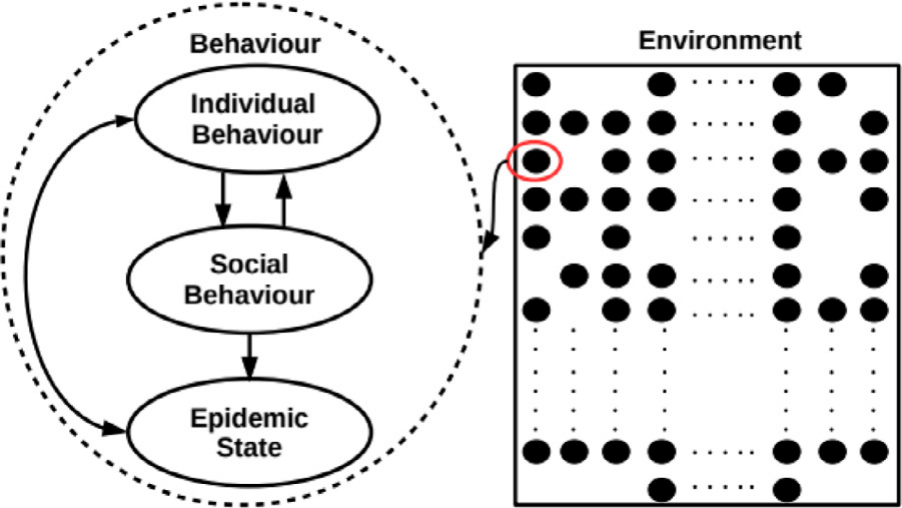
Different levels of behaviour and their relationship are shown at left and the individual's environment within the individuals is shown at right.

Following the idea proposed by Gross [1], we propose a modelling framework based on adaptive coevolutionary networks where the interplay between the dynamic of the epidemic process and the temporal evolution of the structure of the network, that model the social interactions, is explicitly considered. The proposed framework is implemented through an individual-based model built on agents whose behaviour emerges from the interactions of three intertwined components: *i)* Individual behaviour, *ii)* social behaviour and *iii)* health state; whose diagram is shown in Fig. 1. The idea behind this decomposition of the agent's behaviour is to simplify the description of the system and to elucidate the interrelationship between individual behaviour and disease spreading. The modular structure employed to implement it allows to use different mathematical and computational tools for each component, such that the most adequate ones to the problem considered can be chosen.

***Individual behaviour*** determines how an individual reacts to the factors that influence their behaviour like communication, cultural norms, personal circumstances and alternatives, among others. Human behaviour can be influenced by countless factors ranging from media and person-to-person communication to emotions and perceptions [2]. The behaviour towards an infectious disease is determined by a combination of all these factors.

***Social behaviour*** establishes how an individual relates to others. It is given by intraspecific relationships like communication and social practices. It is determined by the individual behaviour but it is also modified by the environment where the individual lies in. This block determines how an individual relates with other individuals. It is related to individual behaviour establishing a feedback relationship. In this context, social behaviour can be modelled through a social network model. This approach gives us a framework where the contacts of the individuals are more relevant than the environment topology.

***Health state*** determines the health evolution of each individual along time. It can be modelled using an automaton that describes the disease evolution through a finite set of epidemiological stages, whose transition can be deterministic or stochastic, or more realistic approaches integrating models of immune response into agents’ dynamic. In this paper we use a finite state automaton that combines both stochastic and deterministic transitions between states, depending on the nature of the disease, improving its modelling capabilities. Furthermore, it allows describing different epidemiological scenarios (quarantine, vaccination, multiple strains, among others), by modifying the automaton (changing the states and transitions), and including individual heterogeneity (modifying the parameters of each cell of the automaton), while it retains modelling simplicity and accuracy.

**Fig. 2:**
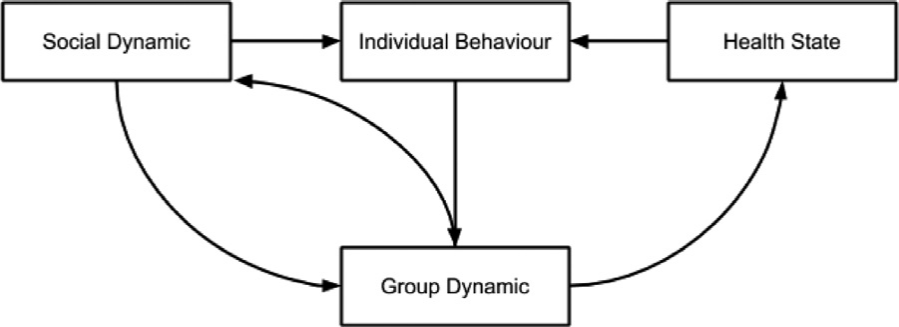
Evolution of the network structure as a consequence of the interaction between different model's blocks.

There is a feedbacked connection between social and individual behaviour since each individual interacts with other members of the group. Thus, it modifies its perceptions and experiences making him react in ways that modify the group dynamic. The presence of a disease modifies the behaviour of each individual, which in turn modifies the structures of the group and society, as shown in Fig. 2.

The evolution of the social group (network topology) depends on the individual's dynamic (nodes). A feedback loop is established between the group (the topology of the network close to each individual) and society (the topology of the entire network) as well as the dynamic between groups.

## Implementation

The *individual behaviour* is modelled through a fuzzy cognitive map (FCM) based on the model proposed by Mei et al. [3]. In this model the concepts C_i_ with *i* *=* 1,2,…,10, are divided into three groups:

• *Input concepts*, where C_1_ is the density of near infected individuals, C_2_ is the density of near recovered individuals and C_5_ is knowledge of the global epidemic situation, representing the perceptions that the agent receives from the environment (primary emotions);
• *Internal concepts***,** representing emotions and feelings of the individual, ie, secondary emotions, where C_3_ is the health state of individual (given by the health state block), C_4_ is knowledge of local epidemiological situation, C_6_ is the assessment of local and global epidemiological situation, C_7_ is optimism level, C_8_ is the memory of similar situations and C_9_ instant reactions;
• *Output concept*, corresponding to the senior emotions, C_10_, representing the actions taken by the agent as a result of a decision process.
• *Inputs u_i_,* where: u_1_, is the density of infected individuals that have contact with a given individual; u_2_ is the density of recovered individuals that have contact with a given individual; u_3_ is the agent current health state and u_4_ is the knowledge of the local epidemiological situation.

**Fig. 3:**
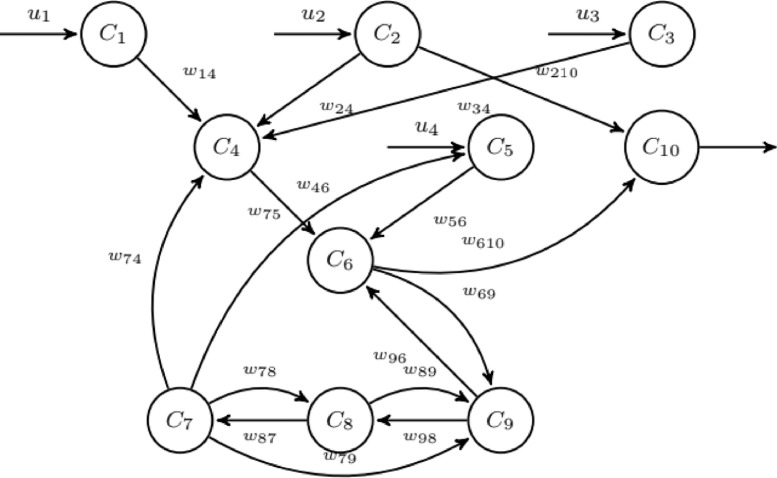
Fuzzy Cognitive Map used to model individual behaviour for flu epidemics. Nodes correspond to the concepts and arrows represent causal relations between concepts.

The value of C_10_ limits the number of contacts made by each individual within the contact space, which affects the effective contact rate. The values of the weights w_ij_ of the matrix W in implementation. The values of the input concepts C_1_ and C_2_ are estimated at each iteration as the density of infected individuals neighbourhood size (*ν*) and density of recovered over neighbourhood size respectively. The value of the concept C_3_ is given by entry u_3_ and is determined by the exit of the Moore machine that models the health state of the individual. In Fig. 3 the graph diagram can be shown

*Social behaviour* is modelled through a network-based model. A *N*×*N* grid defines the contact space where each cell *v_ij_* ∈ V represents a subspace that may be occupied by an individual in any of the possible health states or it is empty. Each cell within the grid corresponds to a node in the network, or an element of the set V. It is possible to simulate the movement of individuals through the reciprocal change of states between adjacent elements in the contact space, ie a cell in the state E_1_ passes to E_2_ while the adjacent cell passes from state E_2_ to E_1_. These movements seek to model the pattern of contacts of individuals and they accomplish a homogeneous distribution of contacts when the contacts of individuals are limited to their neighbourhood. Changing the neighbourhood where an agent can interact with other agent's, the distribution of contacts can be modified, obtaining more realistic contact network topologies. The movement patterns of individuals in the space of the contacts have also a direct effect on the topology, playing an important role in the structure of the contact network.

The function L_t_:V_t_ → V_t_ x X^' *^ established the rules that assign to each element of *V_t_*, the nodes in the contact space, a list of elements (links) of set *X*^'*^. The rules assign the topology *T* to the network at each time *t*. For a square grid and a Moore like the neighbourhood, the number of links assigned to each element of the network is *X_t_* = (2 *r* + 1)^2^ - 1, where r is the radius of the neighbourhood. This means that the radius used determines the degree of each node in the network of contacts. In this way, if you want to model local contacts, the radius not only determines the degree of the node but also limits the area where the node can connect. If you want to model a homogeneous contacts network, like in population-based models, the radio limits the number of contacts of a node but not its space within the network of contacts. Connections to other nodes at each step t are assigned randomly, in order to mimic the random contact hypothesis of compartimental models.

Connections *C* are bidirectional, isotropic and equal at any point of the neighbourhood, providing inputs to each node in state, S. These inputs consist of a value of *λ* that has the node in state I or A, used to make the probabilistic transition from state E. S nodes are included in several connections with infectious individuals in time t will have as many opportunities to change state as the number of contacts. Then, the network is defined as *N=(T, C)*.

It is important to note that the neighbourhood radius in the social behaviour model should not be interpreted as the area of influence of the node. It rather refers to the degree of connectivity of the node in the network. In this way, if we want to simulate local contacts, the radius determines both the area and degree. On the other hand, if we wanted to simulate wide-grid contacts while maintaining the degree of the node, ie number of contacts but not the influence area.

**Fig. 4:**
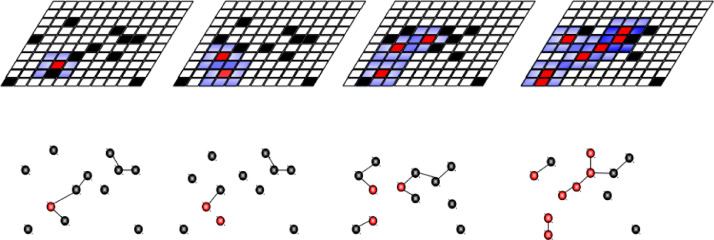
Time evolution of the social network. The radius defines the influence area and the node degree is shown at the top shows the grid of cellular automata in different time *t*, at the bottom you can see how the corresponding network evolves for a particular section of the grid.

For simplicity, the grid used for the construction of the automaton is rectangular, and the neighbourhood is Moore kind with size ν that can change. However, it is possible to use other types of grids and neighbourhoods. These changes in the degree of connectivity between nodes can be seen in Fig. 4. The boundary condition is fixed, with a contour composed of empty non-interacting cells. However, any other boundary condition can be used to model a particular situation.

**Fig. 5:**
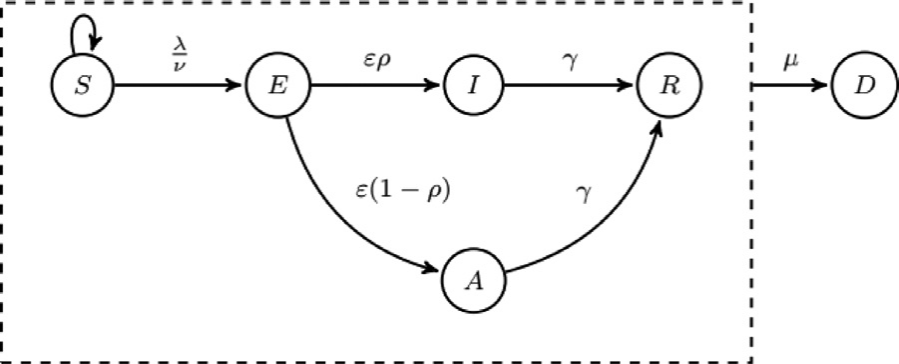
State graph of the health states model. Each node corresponds to an epidemic state and the arrow to the transition between them.

The *health state* is defined as a Moore Machine given by *A= (X,U, E, R*, Λ, *P_0_)*, where the finite states set is defined *X* ={S, E, I, A, R, D}, comprising six epidemic states: S (susceptible), E (exposed), I (infectious symptomatic), A (infectious asymptomatic), R (recovered) and D (dead or empty). *U* ∈ *R* is the input set (Fig. 5).

An individual only receives one active input when it is in state S, emitted by another individual in-state I or A when they are connected in the network. The state transition function is defined as *R: X → X*, applied to the active state at time *t* to probabilistically decides the active state at time *t* *+* *1*. The function is applied in two steps, one corresponding to the change of states by infection and recovery and another corresponding to the movement. If movement is not simulated, then the second step is not performed. In order to decide the state changes two probability matrices are defined, one for the transition on to empty input, where *μ* is the probability of natural death, *γ* is the probability of recovery, and another for the transition by contact with infectious (Table 1) in which taking into account the neighbourhood size *ν* and the input value as *λ* ∈ *U. E* is the output set, the output is non-zero when the state of the node is I or A. Potentially infectious contact occurs between infectious individuals (symptomatic and asymptomatic) and susceptible individuals.

**Table 1:** Transition matrix by contact.

	S	E
S	1- λ/*ν*	λ/*ν*
E	0	1

The output function Λ: *X* → *R* generates the infection rate of the automata if it is in I (*β*) or A (*q β*, being q the probability of infection by contact with an asymptomatic individual). This value is related to the *β'* of the classic model but it is not exactly the same, it is the probability of transmission by contact. The potential number of infectious contacts c is related to the size of the neighbourhood that determines the connectivity with other nodes in the network. In order to obtain the equivalence between the two methodologies, the number of contacts in the network is multiplied by the density of cells occupied in the grid (which may vary according to the grid size used and the number of individuals to be simulated). The combination of the *β'* probability with the potential number of contacts *c* is what will result in the infection rate of each infectious individual. As the value obtained from the parameter setting, the *β* value is obtained from the choice of the neighbourhood size and the *β*' parameter used in the classical model, using the *β* = *β*' c.

The initial state vector *P_0_* ‹S_i_/G_T_,E_i_/G_T_,I_i_ /G_T_,A_i_/G_T_,R_i_ /G_T_› indicates the probabilities of each state being the initial state of the automaton. Defining as G_T_ the total number of cells in the grid and S_i_, E_i_, I_i_, A_i_, R_i_, D_i_ as the initial number of individuals in each state in the grid (Its sum being equal to G_T_ and not to the total population, since D_i_ includes the empty cells). In our case, we only consider probabilities for the states S, E, I and D, derived from the initial values of individuals in the classical model, and the cells that are left empty accordingly. All other states do not have initial individuals.

## Application

We analyse as a study case the Spanish flu in the Swiss canton of Geneva in *1918* [4]. We estimate the model parameters following a two-step procedure: *i)* using a global stochastic optimization method more precisely simulated annealing [5] and then ii) gradient-based local optimization algorithms [6]. The procedure allows us to explore the entire parameter space looking for good candidates (global search), which are used to find the best parameters for the model through a local search. Stochastic optimization methods provide good starting points for gradient-based optimization methods. The objective function used was the normalized squared error (NMSE)

(1) $$\text{NMSE}=\frac{\sum ∥m_{I}\left(k\right)-d_{I}\left(k\right){∥}_{2}^{2}}{∥m_{I}\left(k\right){∥}_{2}^{2}}$$

where m_I_(k) are the predicted value of infected individuals, obtained by the model, and d_I_(k) are the data from the flu epidemic. The resulting parameters are shown in Table 2

**Table 2:** Parameters of the model.

*β*	*ρ*	γ	α	*q*	*N_e_*	*N_i_*	*r*
8.3	0.087	0.246	0.465	0	207	136	3

The *FCM* was trained using Algorithm 1. The value of each concept is calculated taking into account the influence of other concepts over the specific concept taking into account the value of causal relationships between them. As we are modelling a local outbreak (city-level) the value of *C_5_=0.5*, which is equivalent to a phase 4 alert according to the World Health Organization. It is characterized by verified human-to-human transmission at community-level.

**Algorithm 1:** FCM training.

***1)***$W_{0}=w_{ji}\in \left[-1,1\right]$, Set initial weight matrix.	
***2)***$C_{i}^{0}=x_{i}^{0}\in \left[0,1\right]$, Set initial concepts setting.	
***3)***$x_{i}^{t}=f\left({}_{j=1,ji}^{n}x_{j}\left(t-1\right)w_{ji}\right)$, Calculate the value of the concepts.	
***4)***$w_{ji}^{t}=$	$0\;\mathrm{if}\;w_{\mathrm{ji}}^{t-1}=0$
	$\xi w_{\mathrm{ji}}^{t-1}+\eta x_{i}^{t-1}(x_{j}^{t-1}-|w_{\mathrm{ji}}^{t-1}\left|x_{i}^{t-1}\right),\;\mathrm{if}\;w_{\mathrm{ji}}^{t-1}\neq 0,$
***5)* if**$w_{ji}^{t}>1$, **then**$w_{ji}^{t}=1$. **endif** ***6)* if**$w_{ji}^{t}<-1$, **then**$w_{ji}^{t}=-1$, in order to keep $w_{ji}^{t}\in \left[-1,1\right]$ **endif**	
***7)* if**$F1=\left[2\right]C_{10}^{t}-C_{10}^{expected}$ = TRUE, **else if**$F2=|C_{10}^{t+1}-C_{10}^{t}|<\varepsilon \approx 0$= TRUE, **then** exit **else** back to step 2. **endif**	

The resulting matrix *W* is

$$W=\left|\begin{array}{cccccccccc} 0 & 0 & 0 & 0.34 & 0 & 0 & 0 & 0 & 0 & 0\\ 0 & 0 & 0 & -0.14 & 0 & 0 & 0 & 0 & 0 & -0.34\\ 0 & 0 & 0 & 0.44 & 0 & 0 & 0 & 0 & 0 & 0\\ 0 & 0 & 0 & 0 & 0 & 0.52 & 0 & 0 & 0 & 0\\ 0 & 0 & 0 & 0 & 0 & -0.05 & 0 & 0 & 0 & 0\\ 0 & 0 & 0 & 0 & 0 & 0 & 0 & 0 & 0.85 & 0.37\\ 0 & 0 & 0 & -0.13 & -0.27 & 0 & 0 & -0.03 & -0.25 & 0\\ 0 & 0 & 0 & 0 & 0 & 0 & -0.21 & 0 & -0.07 & 0\\ 0 & 0 & 0 & 0 & 0 & -0.14 & 0 & 0.09 & 0 & 0\\ 0 & 0 & 0 & 0 & 0 & 0 & 0 & 0 & 0 & 0 \end{array}\right|$$

**Fig. 6:**
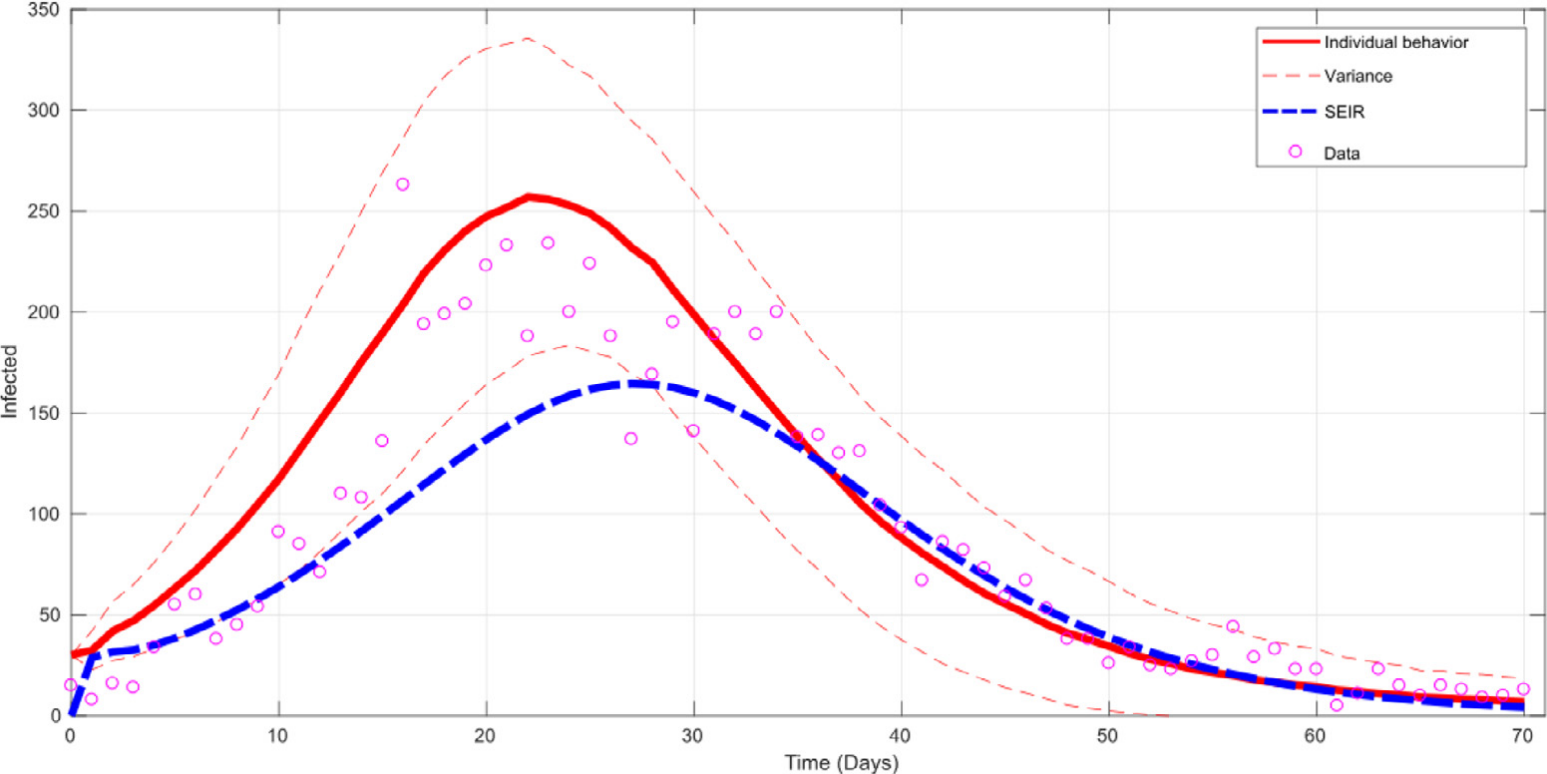
Mean model fitting (solid red line) with variance (dashed red lines) and classic population-based mode (dashed blue line). (For interpretation of the references to colour in this figure legend, the reader is referred to the web version of this article.)

Fig. 6 shows the responses of the proposed and a SEIR models, compared with the real data. The SEIR model is able to capture the initial and final behaviour of the epidemic, however fails to predict the value and time of occurrence of the epidemic peak. On the other hand, the proposed model is able of capturing the epidemic behaviour along the entire process, including the time and magnitude of the infected peak. The normalised mean-square error (NMSE) calculated for the classic SEIR model is 3.3, while the proposed model is 1.6.

## Algorithms

In Algorithm 2 can be seen how given the initial configuration of the contact network, the evolution of the system depends on the interaction of each of the blocks that model the behaviour. At first, the number of contacts of each individual is determined by the *FCM*, then a re-mapping of his contact network through the extraction and replacement algorithms is made and then the health state is updated.

**Algorithm 2:** Model implementation.

G=M_MxN_ MxN square grid where automate evolve
N_T_ Total individuals.
N= Total days.
G=I Initial configuration of the network. (Algorithm 3).
**for***t=1 → N*:
**for** j*=1 → N_T_*:
Individual behaviour actualization j
Health state actualization and Reconfiguration of the contact network of j
**endfor**
**endfor**

It can be seen that for each individual in the network each one of the behavioural blocks is performed. First of all the initialization of the whole system is made as shown in Algorithm 3.

**Algorithm 3:** Initialization algorithm.

G=M_MxN_ (R), Contact network is defined
N_S_= Number of susceptible individuals.
N_I_= Number of infectious individuals.
N_E_= Number of exposed individuals.
N_R_= Number of recovered individuals.
**for i →N_S_:**
G(x,y)=S,1≤x≤M,1≤x≤NSusceptible individual is distributed in the network
**endfor**
**for i →N_I_**:
G(x,y)=I,1≤x≤M,1≤x≤NInfectious individual is distributed in the network
**endfor**
**for i →N_E_**:
G(x,y)=E,1≤x≤M,1≤x≤NExposed individuals are distributed in the network
**endfor**
**for i →N_R_**:
G(x,y)=R,1≤x≤M,1≤x≤N Recovered individual is distributed in the network
**endfor**

In the individual behaviour actualization step the FCM concepts are updated giving the input concepts C_1_, C_2_, C_3_ and C_5_. The new concept values are computed according to Eq. 3

$$C_{i}\left(t\right)=f\left(k_{1}{\sum_{j=1;,j\neq i}^{n}}_{}^{}C_{j}\left(t-1\right)w_{\mathrm{ji}}+k_{2}C_{i}\left(t-1\right)\right),$$

where *k*_2_ is the contribution of the previous value of the concept in the calculation of the new concept and k_1_ is the influence of the interconnected concepts in the configuration of the new value of the concept x_i_. The parameters k_1_ and k_2_ satisfy *0 < k*_1_, *k*_2_ *< 1* and the threshold is $f\left(C_{i}\left(t\right)\right)=\frac{1}{1+e^{-0.3C_{i}\left(t\right)}}$.

The health state actualization is performed in the sub-network extraction step (Algorithm 4), for each individual in the network, Algorithms 6–7 are performed. Thus, it's important to see that the health state configuration is performed before the reconfiguration of the contact network is performed. An Individual's health state is calculated in each reconfiguration stage during the individual's contact actualization.

**Algorithm 4:** Extraction algorithm.

*Ge*=*M*_mxn_(R) ∈ *G*=*M*_MxN_(R) define the extraction sub-net
**for***g_i_* ∈ *G_e_*
**Algorithm 6**
**Algorithm 7**
**Algorithm 8**
**Algorithm 9** (if the individual movement is simulated)
**endfor**

In the reconfiguration of the contact network step, Algorithms 4 and 5 are performed in each time step *t*. The initial configuration of the network (Algorithm 3) is fixed at *t* *=* *0*. The individual's movement is performed by Algorithm 9.

**Algorithm 5:** Replacement algorithm.

*Gr*=*M*_mxn_(R) ∈ *G*=*M*_MxN_(R) defines the segment of the network to replace
*Gr= Ge*

**Algorithm 6:** Infectious state.

**if** State=I
**if** Neighbor=S **then***Z* ~ *U*[0, 1]
**if***Z* < *β*/*ν***then** State=E
**endif**
**endif**
**Endif**
**if** State=*A*
**if** State=S **then***Z* ~ *U*[0, 1]
**if***Z* < *qβ*/*ν***then** State=E
**endif**
**endif**
**endif**

**Algorithm 7:** Exposed state.

**if** State=*E***then***Z* ~ *U*[0, 1]
**if***Z* < ɛ*ρ***then** State=*I*
**if**Z<ε(1−ρ)**then** State=A
**endif**
**endif**
**endif**

**Algorithm 8:** Recovery phase.

**if** State=*I***or** State=*A***then***Z* ~ *U*[0, 1]
**if** Z < γ **then** State=R
**endif**
**endif**

**Algorithm 9:** Individuals movement.

Z1,Z2∼U[−r,r]
Aux=State(i,j)
State(i,j)=State(i+Z_1_,j+Z_2_)
State(i+Z_1_,j+Z_2_)=Aux

## Method validation

In this section, we carry out an exhaustive numerical analysis in order to validate the proposed model and compare it with the classic model proposed by Chowell [4] and the model without individual behaviour, which makes it a cellular automaton [7].

The models were numerically validated using the *Akaike Information Criterion* (*AIC*), which provides a measure of model quality considering both accuracy and complexity simultaneously. This approach is widely used to measure the quality of models and validate them [8]. This criterion is equivalent to a cross-leave-one-out in longitudinal data model validation [9]. Models that have an *AIC* within the range 1–2 consistently support structural variation in the data. Those models that have their value in the range 3–7 withstand significantly structural variation in the data. Finally, those models that have *AIC >* 10 do not explain structural changes in the data. The *AIC* index is computed as is shown

(2) $$\mathrm{AIC}=\log\left(\det\left(\frac{1}{m}\sum_{1}^{m}\varepsilon \left(t,\Theta \right)\varepsilon {\left(t,\Theta \right)}^{T}\right)\right)\frac{2n}{m}$$

where Θ is the set of *n* estimated parameters *m* is the number of samples and ɛ(*t*, Θ) is the measured error. The error measure used for this analysis was the raw residuals calculated as $r=y_{i}-\hat{y_{i}}$, where *y_i_* is the model output (infected population) and $\hat{y_{i}}$ is the real data. The *AIC* was used instead of *Bayesian Information Criterion BIC* because the first one is an estimate of the relative distance between the true dynamic function and the model dynamic, plus a constant, while the *second* is an estimate of the posterior probability of a model considered true under some Bayesian configuration. For both the model with and without individual behaviour, the same value of *n* (*n* *=* 8) is considered since the parameters that govern the *FCM* are fitted in an independent process that is detailed later in this section. For the classic model the number of fitted parameters is 11. The number of samples considered for the fitting process was 70 (daily).

The statistical significance of these results were assessed by computing the probability of error of both models. The results show that the proposed model is better than the *SEIR* and the model without individual behaviour (see Table 3). For this test, statistical independence of errors adjustment for different data sets is assumed and the errors of a binomial distribution are approximated with a Gaussian distribution. One of the fundamental aspects of the model evaluation is to estimate the probability of error, because on one hand it allows us to evaluate the usefulness of the model for the intended purposes and on the other hand allows us to compare their performance against other models.

**Table 3:** Statistical measures of raw residuals for the different models, where $\bar{X}$ is the mean, M the mode, σ is the standard deviation, Kurt is the kurtosis and AIC is the AIC index.

Method	$\bar{X}$	M	σ	Kurt	AIC
Classic approach [4]	−19.57	−9.15	54.75	1.78	7.5
Individual behaviour	4.5	−2.08	45.87	1.75	6.1
No Individual behaviour	−5.8	−3.13	45.5	1.76	6.5

Three new data sets were generated with gaps randomly chosen with uniform probability: *i)* a data set with *n* *=* 10 data points removed (14% of the original data set), *ii)* a data set with *n* *=* 20 (28% of the original data set) data points removed and *iii)* a data set with *n* *=* 30 data points (43% of the original data set). All the methods were fitted with each data set to get seven generations of parameters for each one. Then, we perform 1000 simulations for each generation of parameters obtained in the previous step in order to obtain a good approximation of the average response. Finally, the average error for each is calculated taking into account the average response model using data that was extracted from the original data set. Thus for each set of fitted parameters with different gaps in the data set that the proposal we hypothesize that *P*(*Error_method_ < Error_alternative_*) *> p*.

Table 4 shows the results for statistical significance for the different validation sets. It can be seen that the proposed model is better than the classic SEIR [4], having confidence intervals error above 99%. Table 5, on the other hand, shows the results for statistical significance for the different validation sets between the proposed method (with individual behaviour) and without individual behaviour, having confidence intervals error above or near to the 90%*.*

**Table 4:** Significance of the error for the model for different data-set training size. *MSE is the error measure used for the test*.

10 gaps data set				
Model	*MSE*	*μ*	σ	*P*(*p_1_<p_2_*)
Proposed method	7.65	0.24	0.0043	99.5
Classic approach [4]	10.4	0.896	0.0050	

20 gaps data set				
Model	*MSE*	*μ*	σ	*P*(*p_1_<p_2_*)
Proposed method	4.5	0.955	0.0034	99.7
Classic approach [4]	5.75	0.943	0.0038	

30 gaps data set				
Model	*MSE*	*μ*	σ	*P*(*p_1_<p_2_*)
Proposed method	4.3	0.957	0.0033	99.9
Classic approach [4]	5.88	0.941	0.0038

**Table 5:** Significance of the error for the model for different data-set training size. *MSE is the error measure used for the test*.

10 gaps data set				
Model	*MSE*	*μ*	σ	*P*(*p_1_<p_2_*)
No individual behaviour	8.09	0.919	0.0044	80.6
Individual behaviour	7.56	0.924	0.0043	

20 gaps data set				
Model	*MSE*	*μ*	σ	*P*(*p_1_<p_2_*)
No individual behaviour	5.01	0.950	0.0035	80.78
Individual behaviour	4.66	0.953	0.0034	

30 gaps data set				
Model	*MSE*	*μ*	σ	*P*(*p_1_<p_2_*)
No individual behaviour	4.9	0.951	0.0035	88.84
Individual behaviour	4.3	0.957	0.0033	

**Fig. 7:**
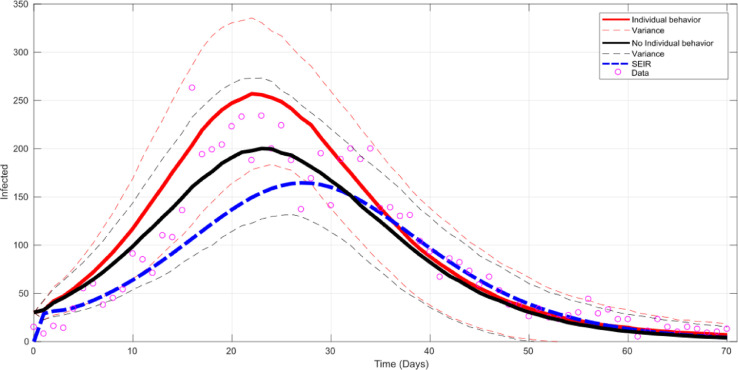
Model fitting with individual behaviour (solid red), without individual behaviour (solid black) and classic SEIR (dashed blue). (For interpretation of the references to colour in this figure legend, the reader is referred to the web version of this article.)

As a visual validation of the method, Fig. 7 shows the mean fitting of the model with individual behaviour (solid red curve) and its respective variance (dashed red lines), the mean fitting of the model without individual behaviour, that is without FCM, (solid black curve) with its variance (dashed black curve) and the mean fitting of the classical model (dashed blue line). The inclusion of individual perception (with FCM) tends to reproduce the real dynamics in a very accurate way. This can be seen in the exponential phase of the epidemic and also at the peak of the outbreak where the model captures the average behaviour very well despite the dispersion of the data.

**Fig. 8:**
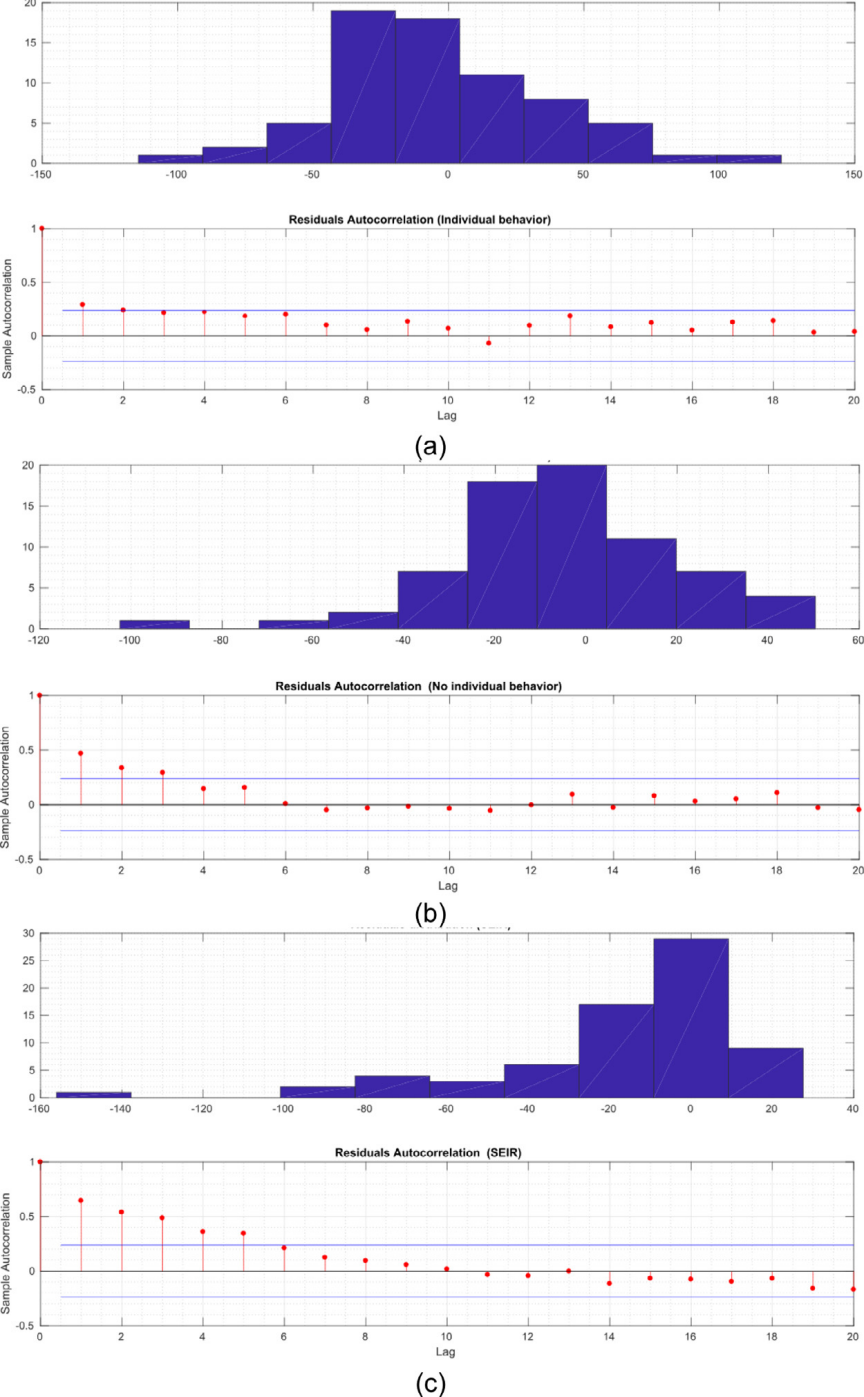
Raw residuals distribution (upper panel) and autocorrelation for the proposed method with individual behaviour (a), without individual behaviour (b) and a classic SEIR approach (c).

Fig. 8 shows the distribution of the residuals for each of the models. Fig. 8a the distribution of the residuals for the model with individual behaviour, which follows a quasi-normal distribution and the autocorrelation suggests that there is no dependency for lags above *2*. Similar behaviour can be observed for the individual-based model behaviour (without FCM) although the distribution of the residuals deviates a little from the normal form and the dependence for lags less than 4 in the autocorrelation coefficients suggests some structural dependence (Fig. 8b). In the case of the classical model, the behaviour described above is not observed (Fig. 8c).

### Individual behaviour

For the individual's behaviour actualization step the *FCM* concepts are updated giving the input concepts *C_1_, C_2_, C_3_* and *C_5_*. The new concepts are computed according to Eq. (3).

(3) $$C_{i}\left(t\right)=f\left(k_{1}{\sum_{j=1;,j\neq i}^{n}}_{}^{}C_{j}\left(t-1\right)w_{\mathrm{ji}}+k_{2}C_{i}\left(t-1\right)\right)$$

where *k_2_* is the contribution of the previous value and *k_1_* is the influence of the related concepts. The two parameters *k_1_* and *k_2_* satisfy 0 <*k_1_, k_2_*<1 and *f* is the threshold.

Algorithm 1 was used for the *FCM* training. For the four step rule, *η* ≈ 0 is the learning coefficient, ξ is the loss of the learning coefficient. *F1* y *F2* are the termination criteria. *F1* is the minimization for the Euclidean distance between the current value of the concept of output and the expected value. Taking into account that $C_{10}\in \left[C_{10}^{\text{min}},C_{10}^{\text{max}}\right]$, the value $C_{10}^{expected}$ must be $C_{10}^{expected}=\left(C_{10}^{\text{min}}+C_{10}^{\text{max}}\right)/2$. The second rule is used to ensure the convergence of the method after a number of iterations, being ϵ≈0.

The threshold function is shown in Eq. (4). This kind of function is particularly useful if when you know the range where *x* can take values during the inference process [10] thus, the output is not limited to a specific range as with other kinds of threshold functions such as a sigmode [3,11].

(4) $$f\left(x\right)=\frac{1}{2}\left(\alpha x+1\right),$$

where $\alpha =\frac{0.5}{\rho_{1}+\rho_{2}∥W∥}n^{1/2}$, and

*ρ*_1_∧*ρ*_2_ ∈ [0, 1] and *W* is the weight matrix.

**Fig. 9:**
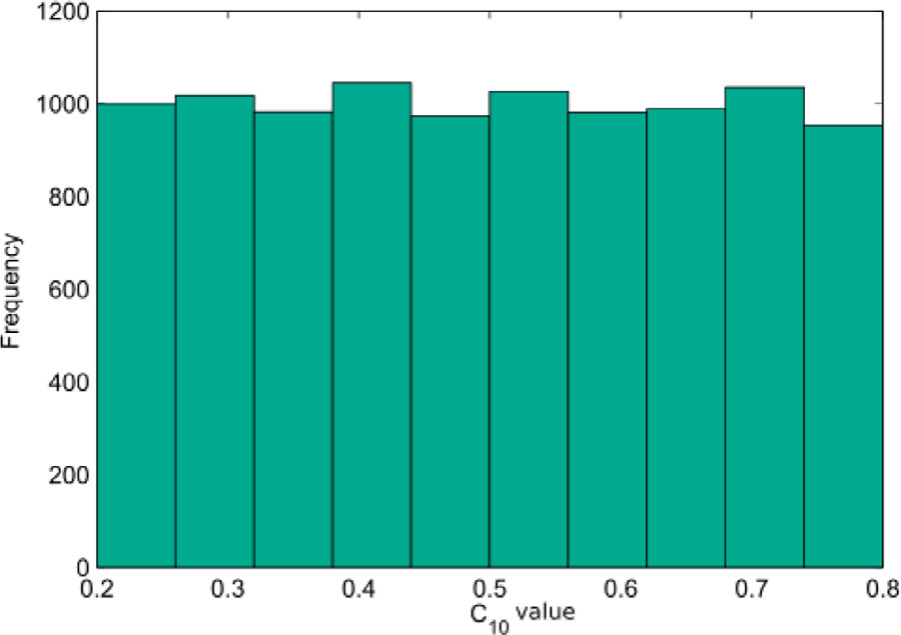
Output concept (*C_10_*) distribution during the training process.

To validate the weight matrix *W* a set of 10,000 vectors *X_0_* was generated, where each $x_{i}\in \left[0,1\right],i=1,...10$ using an uniform distribution, then the computation of C_10_ was performed using Eq. (3). The results re inside the interval [0.2,0.8] (Fig. 9).

## Declaration of Competing Interest

The authors declare that they have no known competing financial interests or personal relationships that could have appeared to influence the work reported in this paper.
